# Correlation of cervical sagittal alignment parameters on full-length spine radiographs compared with dedicated cervical radiographs

**DOI:** 10.1186/s13013-016-0072-0

**Published:** 2016-04-07

**Authors:** Leah Y. Carreon, Casey L. Smith, John R. Dimar, Steven D. Glassman

**Affiliations:** Norton Leatherman Spine Center, 210 East Gray Street, Suite 900, Louisville, KY 40202 USA; Central States Orthopedic Specialists, William Medical Building, 6585 S. Yale Ave. Ste. 200, Tulsa, OK 74136 USA

**Keywords:** Cervical spine, Adult spinal deformity, Sagittal parameters

## Abstract

**Background:**

Radiographic parameters to evaluate the cervical spine in adult deformity using 36-inch films have been proposed. While 36-inch films are used to evaluate spinal deformity, dedicated cervical films are more commonly used to evaluate cervical spine pathology. The purpose of this study is to determine correlations between sagittal measures from a dedicated cervical spine radiographs and 36-inch spine radiographs.

**Methods:**

Patients who had standing cervical and 36-inch radiographs within four weeks of each other were identified. On separate occasions, the following measures were determined: C0-C2, C0-C7, C1-C2 and C2-C7 sagittal Cobb angles; T1 slope; chin-brow-vertical angle (CBVA), C1-C7 sagittal vertical axis (SVA), C2-C7SVA, center of gravity-C7 sagittal vertical axis (COG-C7SVA). Paired t-tests and correlation analyses were done between parameters from the cervical and the 36-inch film.

**Results:**

Radiographic measurements were collected on 40 patients (33 females and 7 males, mean age of 48.9 ± 14.5 years). All correlations were statistically significant at *p <* 0.001. C0-C2 Cobb had the strongest correlation (*r =* 0.81) and C2-C7 Cobb had the weakest (r=0.62). Among sagittal balance parameters, COG-C7SVA had the weakest correlation (*r =* 0.42) and C1-C7SVA (*r =* 0.64) and the C2-C7SVA (*r =* 0.65) had strong correlations. The T1 slope and the CBVA had correlation coefficients of 0.74 and 0.91, respectively. There was no statistically significant difference in measures taken from the cervical film and 36-inch film, except for the C0-C7 Cobb (*p =* 0.000) with a measurement difference of 7° and the T1 tilt (*p =* 0.000) with a measurement difference of 5°.

**Conclusion:**

Except for COG-C7 SVA, strong correlations between most cervical spine parameters taken from a dedicated cervical film and those taken from a 36-inch film were seen. 36-inch radiographs provide a reasonable estimation of cervical sagittal spine parameters and may obviate the need for a dedicated cervical spine radiograph.

## Background

Over the past 10 years, there has been an increased focus on the evaluation and treatment of adult scoliosis [1–3]. Several studies have examined the complexity of adult scoliosis patients, based in part on the interaction of the deformity with the normal aging processes of the spine [2–7]. The intersection between degeneration and deformity is most evident in relation to the lumbar spine. Typically, assessment of adult scoliosis patients involves evaluation of both the primary deformity and the lumbar spine [4, 8, 9]. Even when managing a primary thoracic curve, treatment decisions may revolve around the impact of any potential surgery on the unfused lumbar levels [4, 8–11].

Recently, attention has been directed to the impact of adult scoliosis or scoliosis treatment on the cervical spine [12–20]. Several authors have proposed a set of standardized radiographic parameters [21] to help evaluate the cervical spine in patients with adult spinal deformity using full-length 36-inch radiographs. While this is the standard radiograph used to evaluate spinal deformity, dedicated cervical spine radiographs are more commonly used to evaluate cervical spinal pathology.

With the need to limit costs and exposure to radiation, there is a need to determine whether a separate cervical spine radiograph, aside from the long 36-inch radiograph, is necessary to evaluate the sagittal parameters of the cervical spine. Recent studies have reported a higher incidence of cancer in adolescent idiopathic scoliosis patients who have had multiple radiographs [22]. As the effect of radiation exposure is cumulative, decreasing the number of radiographs taken over an individual’s lifetime, regardless of age, should be considered. The purpose of this study is to determine whether there is a correlation between sagittal measures of the cervical spine taken from the 36-inch spine radiographs and sagittal measures of the cervical spine taken from cervical spine radiographs.

## Methods

From a multi-surgeon spine specialty clinic, patients who had a 36-inch spine radiograph as well as a separate standing cervical spine radiograph within four weeks of each other were identified. All radiographs were taken using a Picture Archiving and Communication System (PACS). All 36-inch standing radiographs were taken with the beam centered at the thoracic area in order for both femoral heads and the cervical spine to be visible. All 36-inch spine films were taken in the “clavicle” position [23]. The “clavicle” position has the patient full flex both elbows with the hands in a relaxed fist, wrists flexed, hands are centered in the supraclavicular fossae, midway between the suprasternal notch and acromion, passively flexing the humerus forward. This position has been standard at our center since 2002. All dedicated cervical spine films were taken with the beam centered at C4 approximately the level of the angle of the mandible.

**Fig. 1:**
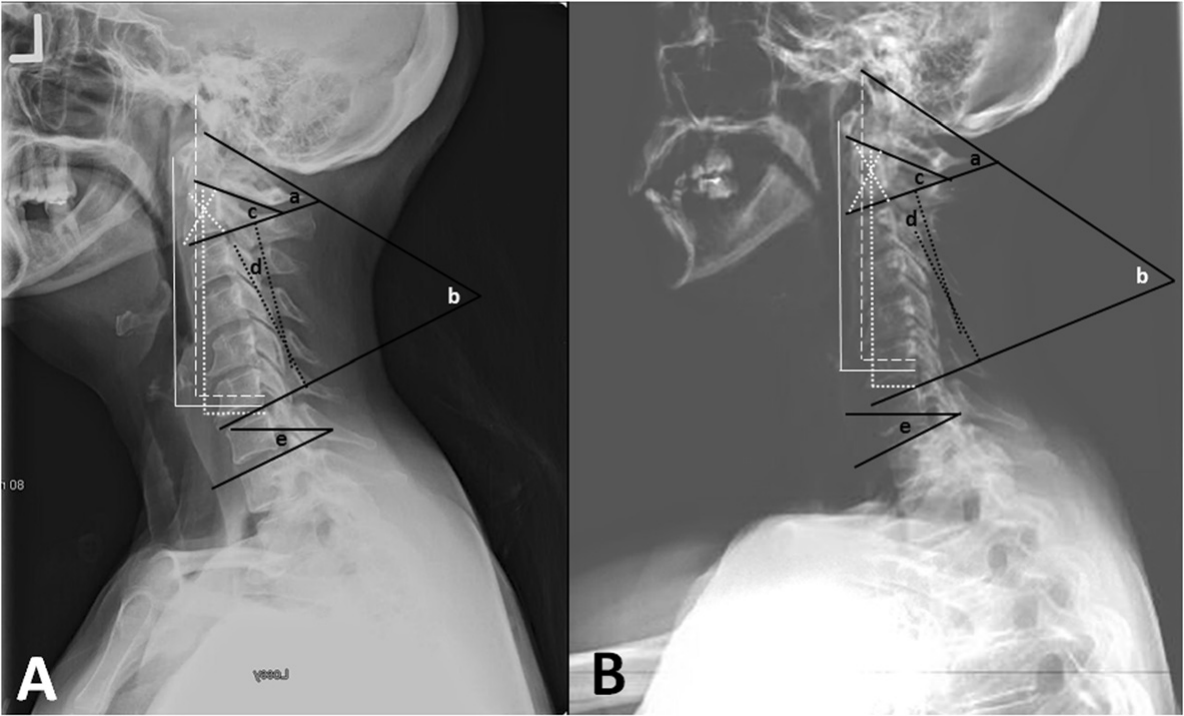
**a** Cervical lateral radiograph; **b** Detail of 36-inch lateral radiograph showing measurements (a) Occiput-C2 sagittal Cobb angle, (b) Occiput-C7 sagittal Cobb angle, (c) C1-C2 sagittal Cobb angle, (d) C2-C7 sagittal Cobb angle, (e) T1 tilt. Horizontal solid white line: C1-C7 Sagittal Vertical Axis - distance between plumb line dropped from anterior tubercle of C1 and posterior superior corner of C7; Horizontal white dotted line: C2-C7 Sagittal Vertical Axis—distance between plumb line dropped from centroid of C2 and posterior superior corner of C7; White dashed line: Center of Gravity-C7 Sagittal Vertical Axis—distance between plumb line dropped from anterior margin of external auditory meatus and posterior superior corner of C7

From the de-identified 36-inch and cervical spine radiographs, the following radiographic measures [21] were determined: occiput-C2 (C0-C2), occiput-C7 (C0-C7), C1-C2 and C2-C7 sagittal Cobb angles (Fig. 1); T1 slope; chin-brow vertical angle (CBVA), C1-C7 sagittal vertical axis (C1-C7 SVA), C2-C7 sagittal vertical axis (C2-C7 SVA) and center of gravity- sagittal vertical axis (COG-C7 SVA). The measurements for the 36-inch radiographs and cervical spine radiographs were done on separate sessions by a single-observer. Standard demographic data including age and sex were also collected.

T1 slope is the angle between the angle between a horizontal line and the upper end plate of T1. Sagittal plane translation of the cervical spine is measured through the C7 SVA, which is a plumb line in line with the posterior superior aspect of C7. C1-C7 SVA is the distance between a plumb line dropped from the anterior tubercle of C1 and the C7-SVA. C2-C7 SVA is the distance between a plumb line dropped from the centroid of C2 (or odontoid) to the C7 SVA. COG-C7 SVA is the distance between a plumb line dropped from the anterior portion of the external auditory canal to the C7 SVA.

Paired t-tests and correlation analyses were performed between the sagittal radiographic parameter as measured on the cervical spine radiograph and corresponding paired radiographic parameter on the 36-inch radiograph. Correlation coefficients between 0.60 and 0.80 indicate a marked degree of correlation; while coefficients between 0.80 and 1.00 indicate robust correlations [24].This study was reviewed and approved by the University of Louisville Institutional Review Board (13.0757) and the Norton Healthcare Office of Research Administration (13-N0234).

## Results

Radiographic measurements were collected on 40 patients. There were 33 females and 7 males with a mean age of 48.9 ± 14.5 years. All correlations were statistically significant at *p <* 0.001 (Table 1). All sagittal Cobb measures showed a marked correlation. The C0-C2 sagittal Cobb had the strongest correlation (*r =* 0.81) and the C2-C7 sagittal Cobb had the weakest (0.62). Among the sagittal balance parameters, the COG-C7 SVA had the weakest correlation (*r =* 0.42), and the C1-C7 SVA (*r =* 0.64) and the C1-C7 SVA (*r =* 0.65) had strong correlations. The T1 slope and the CBVA had correlation coefficients of 0.74 and 0.91, respectively. Paired t-tests showed that there was no statistically significant difference in the measures taken from the cervical radiograph and 36-inch radiograph (Table 2), except for the occiput-C7 sagittal Cobb angle (*p =* 0.000) with a measurement difference of 7° and the T1 tilt (*p =* 0.000) with a measurement difference of 5°.

**Table 1:** Correlation coefficients between radiographic parameters measured on the 36-inch radiograph and the cervical spine radiograph. All correlations were statistically significant at *p <* 0.001

Radiographic parameter	Correlation coefficient
Occiput-C2 Sagittal Cobb angle	0.808
Occiput-C7 Sagittal Cobb angle	0.678
C1-C2 Sagittal Cobb angle	0.639
C2-C7 a Sagittal Cobb angle	0.620
T1 tilt	0.742
C2-C7 Sagittal Vertical Axis	0.653
C1-C7 Sagittal Vertical Axis	0.635
Center of Gravity-C7 Sagittal Vertical Axis	0.415
Chin-brow-vertical angle	0.911

**Table 2:** Mean (standard deviation) of radiographic parameters as measures on the 36-inch and cervical spine radiograph

Radiographic parameter	36-inch	Cervical	*p*-value	Difference
Occiput-C2 Sagittal Cobb angle (degrees)	−41.89 (9.74)	−43.30 (8.70)	0.139	1.40 (5.79)
Occiput-C7 Sagittal Cobb angle (degrees)	−44.94 (11.0)	−51.92 (9.89)	0.000	6.98 (8.44)
C1-C2 Sagittal Cobb angle (degrees)	−33.87 (8.65)	−33.95 (14.78)	0.963	0.09 (11.39)
C2-C7 a Sagittal Cobb angle (degrees)	−4.73 (12.96)	−8.58 (12.81)	0.041	3.86 (11.23)
T1 tilt (degrees)	23.87 (8.82)	28.90 (8.64)	0.000	−5.03 (6.28)
C2-C7 Sagittal Vertical Axis (mm)	15.28 (14.36)	20.04 (18.47)	0.049	−4.76 (14.18)
C1-C7 Sagittal Vertical Axis (mm)	22.14 (18.34)	26.95 (21.02)	0.090	−4.80 (16.99)
Center of Gravity-C7 Sagittal Vertical Axis (mm)	14.23 (14.84)	17.06 (14.80)	0.304	−2.83 (16.03)
Chin-brow-vertical angle (degrees)	−62.79 (5.17)	−61.93 (4.42)	0.098	−0.86 (2.16)

## Discussion

The importance of restoration of sagittal spinal alignment on treatment effectiveness and clinical outcomes during deformity correction has been the subject of numerous studies [9, 25, 26]. Most of these studies focus on the importance of the restoration of lumbar lordosis and its relation to the pelvic incidence [8, 9, 24]. Only recently has the role of cervical sagittal measures in outcomes for spine deformity been studied [12–20].

A study by Smith et al. [27] showed that surgical correction of positive sagittal spinopelvic malalignment results in improvement of abnormal cervical hyperlordosis. In contrast, Oh et al. [16] showed that cervical lordosis is commonly seen in patients with adult spinal deformity and does not appear to normalize after thoracic corrective surgery. Patients with substantial compensatory cervical lordosis have been shown to be at increased risk of sagittal spinal pelvic malalignment [17]. Also, a study on adult spinal deformity patients showed that a more proximal upper end vertebra was predictive of the presence of neck pain complaints [28].

Thus, with the increasing evidence of the role of cervical sagittal parameters on clinical outcomes and disability in adult spinal deformity along with the need to control costs and limit patient exposure to radiation, this study was undertaken to determine whether a separate cervical spine radiograph, aside from the long 36-inch radiograph, is necessary to evaluate the sagittal parameters of the cervical spine. Data from this study showed that, except for COG-C7 SVA, there were strong correlations between most cervical spine parameters taken from a dedicated cervical spine radiograph and those taken from a 36-inch radiograph. In addition, measures taken from the cervical radiograph and 36-inch radiograph were similar, except for the C0-C7 sagittal Cobb and the T1 tilt. Whether these differences have any clinical relevance needs to be further studied. Especially since the only study published looking at cervical sagittal parameters and clinical outcomes showed weak correlations between patient reported outcomes and C2-C7SVA and COG-C7 SVA [29]. In certain patients, dedicated cervical spine films may still be indicated to rule out malignancy or other pathologies. Further studies with multiple observers should also be done to determine the reliability of these cervical measures as determined from a 36-inch radiograph and dedicated cervical spine film.

## Conclusions

A dedicated cervical spine radiograph may not be necessary to evaluate the sagittal parameters of the cervical spine when a full-length 36-inch radiograph has already been obtained.
